# Publisher Correction: Morphodynamics facilitate cancer cells to navigate 3D extracellular matrix

**DOI:** 10.1038/s41598-021-01168-8

**Published:** 2021-10-28

**Authors:** Christopher Z. Eddy, Helena Raposo, Aayushi Manchanda, Ryan Wong, Fuxin Li, Bo Sun

**Affiliations:** 1 Department of Physics, Oregon State University, Corvallis, OR 97331 USA; 2 Department of Chemical, Biological, and Environmental Engineering, Oregon State University, Corvallis, OR 97331 USA; 3 Molecular and Cellular Biology Program, Oregon State University, Corvallis, OR 97331 USA; 4 School of Electrical Engineering and Computer Science, Oregon State University, Corvallis, OR 97331 USA

Correction to: *Scientific Reports* 10.1038/s41598-021-99902-9, published online 14 October 2021

The original version of this Article contained errors in Figure 4E, where additional text was erroneously introduced into the bar graph.

The original Figure 4 and accompanying legend appears below.

**Figure 4:**
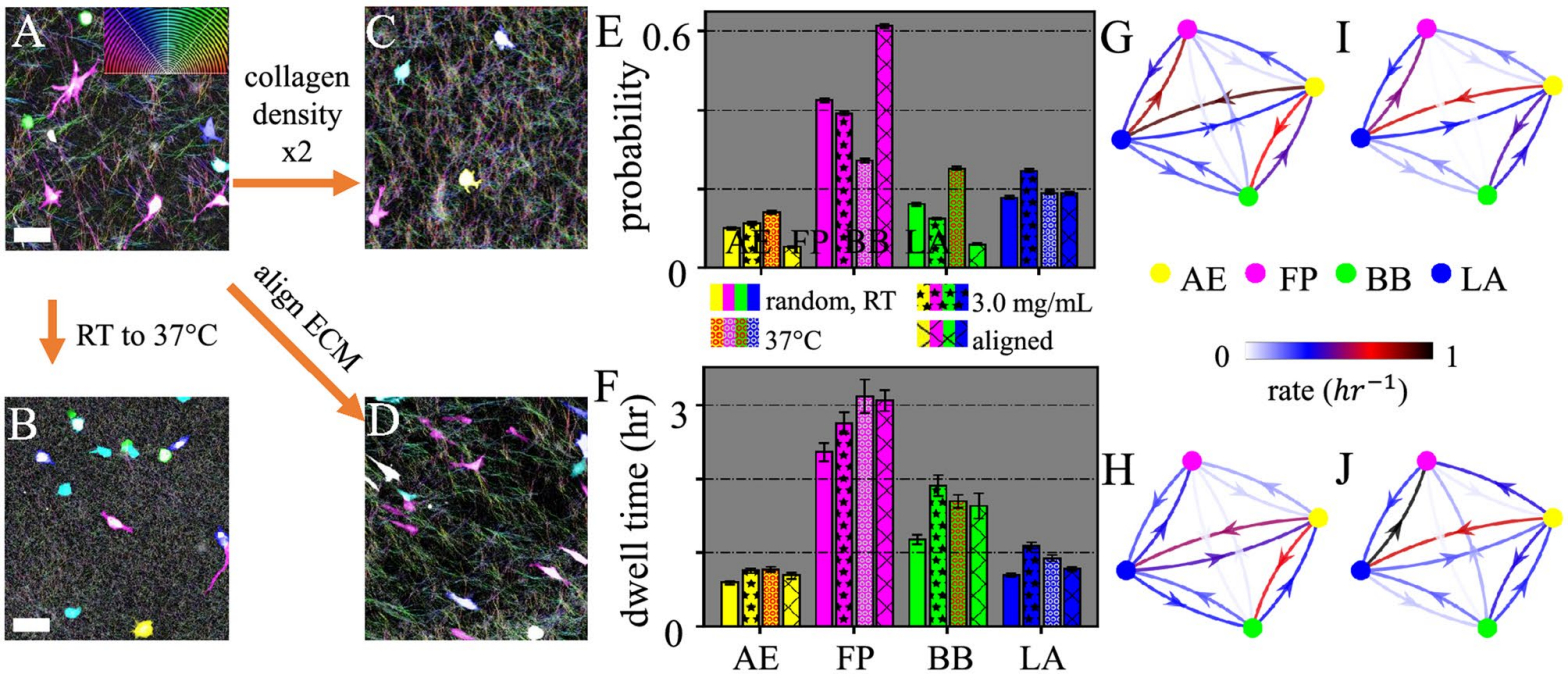
Physical properties of collagen ECM regulate the morphological phenotype homeostasis of 3D migrating MDA-MB-231 cells. (**A**–**D**) Confocal reflection images and pseudo colored MDA-MB-231 cells for collagen matrices prepared at varying conditions. Scale bars: 20 μm. **A** Collagen ECM prepared at room temperature (RT, or 25 °C) and collagen concentration of $[col]=1.5$ mg/mL. **B** Collagen ECM prepared at 37 °C and $[col]=1.5$ mg/mL. **C** Collagen ECM prepared at RT and $\left[col\right]=3.0$ mg/mL. **D** collagen ECM prepared with flow-aligned collagen fibers. (**E**) Fraction of cells in each morphological phenotype. 8000 single cell images are analyzed under each ECM condition. (**F**) Dwell time of cells in each morphological phenotype. Errorbars in (**E**, **F**) represent 95% confidence intervals calculated from 1000 bootstrap iterations. (**G**–**J**) The transition matrix—morphological phenotype transition rates under varying ECM conditions. **G** Collagen ECM prepared at room temperature and $\left[col\right]=1.5$ mg/mL. **H** Collagen ECM prepared at 37 °C and $\left[col\right]=1.5$ mg/mL. **I** Collagen ECM prepared at RT and $\left[col\right]=3.0$ mg/mL. **J** Collagen ECM prepared with flow-aligned collagen fibers. Under each ECM condition a total of more than 2000 h of single cell trajectories are analyzed. This figure is prepared with Matlab R2020a (www.mathworks.com) and ImageJ (https://imagej.net).

The original Article has been corrected.

