# Supplementary material for: Morphodynamics facilitate cancer cells to navigate 3D extracellular matrix
Source: Sci Rep. 2021 Oct 14;11:20434. doi: 10.1038/s41598-021-99902-9 (PMC8516896; doi:10.1038/s41598-021-99902-9)
Supplement: Supplementary file 1 — Supplementary Information 1. [file 41598_2021_99902_MOESM1_ESM.pdf]

Morphodynamics Facilitate Cancer Cells to Navigate 3D Extracellular Matrix
Supplementary Information

Christopher Z. Eddy[1], Helena Raposo[2], Aayushi Manchanda[3], Ryan Wong[1], Fuxin Li[4], and Bo Sun[1,*]

[1]*Department of Physics, Oregon State University, Corvallis OR, 97331*
[2]*Department of Chemical, Biological, and Environmental Engineering, Oregon State University, Corvallis OR, 97331*
[3]*Molecular and Cellular Biology Program, Oregon State University, Corvallis OR, 97331*
[4]*School of Electrical Engineering and Computer Science, Oregon State University, Corvallis OR, 97331*
[*]*Correspondence to sunb@onid.orst.edu*

S1: Additional Experimental Details

3D cell culture

GFP-labeled MBA-MB-231 human breast carcinoma cells are purchased from GenTarget Inc. and are maintained according to the manufacturer's instructions. Briefly, growth media is prepared using Dulbeccos Modified Eagle Medium (Gibco, US) supplemented with 10% fetal bovine serum (Gibco, US), 1% penicillin-streptomyocin (Gibco, US), and 0.1 mM non-essential amino acid (NEAA 100x, ThermoFisher, US). Collagen solutions are prepared by diluting rat-tail collagen type I (Corning, US) with prepared growth medium, phosphate-buffered saline (PBS, 10x), and sodium hydroxide (NaOH, 0.1M) to a concentration of 1.5 mg/mL or 3.0 mg/mL with pH 7.4. To embed the cells in 3D collagen matrices, cells are suspended at very low density of approximately 650 cells/μL in ice-cold neutralized collagen solution and added to a 35 mm collagen coated glass bottom dish with a 7 mm microwell diameter (MatTek, US). The microwell containing ice-cold cell-collagen solution is covered with a coverslip so that the dish may be inverted during gelation to ensure dispersion of cells in 3D. The dish is then incubated on either a warming plate set to 25°C, or in a tissue culture incubator (37°C, 5% CO_2) for 30 minutes in order to solidify the matrix. For fiber alignment, a small magnetic iron shaving (<200 μm) is first placed onto the dish and then immersed in cell-collagen solution and placed onto the warming plate. We then drag the particle through the solution by an external magnet along a line for approximately 3 minutes while warming, and is then left to solidify [1]. The coverslip is removed after gelation time and the cellularized ECM is immersed with tissue culture medium and continuously incubated for 24 hours before imaging.

Immunofluorescence

Following a standard two-step immunofluorescence staining protocol (ThermoFisher), each sample is incubated at room temperature with 4% formaldehyde solution in PBS for 15 minutes to fix the sample. After washing with PBS for an additional 5 minutes, the sample is then incubated at room temperature for 15 minutes in a working concentration of 0.5% Triton X-100 in PBS, used as a permeabilization agent. We then further wash the sample with PBS for 5 minutes. To prevent non-specific binding, a 3% bovine serum albumin blocking solution in PBS is added and then incubated at room temperature for 60 minutes. We then wash the sample with PBS for 5 minutes. F-actin antibody (Invitrogen) or phalloidin dye (Invitrogen) is added and then incubated at room temperature for 30 minutes. Finally, the sample is washed with PBS for 5 minutes and then is imaged with a 40X objective.

Microscopy and image analysis

Continuous imaging is done with a Leica TCS SPE confocal microscope equipped with a stage-top incubator (ibidi). Images are captured at a rate of 1 frame per 15 minutes. The raw images are gray scale with a resolution of 1024 x 1024 pixel^2. The voxel size has been calibrated to equal 0.538 μm. A single x-y plane is imaged every 10 μm in the z-dimension per experiment for up to 24 hours. Using custom Matlab scripts, cell images are maximum-projected onto a x-y plane and tracked over time (see section S2). The projected images are manually segmented, and screened to remove cells that are not entirely within the viewing window.

S2: Geometric Characterization of Cell Images

Image processing

Following acquisition of fluorescent images, data regarding cell shape and position are obtained by processing and binarizing the time-lapse images using custom NIH ImageJ and Matlab scripts. First, fluorescence images are background subtracted using a rolling ball radius of 50 pixels (26.88 μm) and then log-transformed in order to make cell edges highly visible and so that less fluorescent-intense cells are also quantified. A manual threshold is then applied for each image. After, cells are manually segmented for each z-stack if applicable. Since consecutive z-stacks may have cell overlap, custom Matlab scripts are then used to determine if the same cell is in multiple z-stacks. After, we take a maximum projection (2D) of each cell. Geometrical measurements are then taken on binary objects using Matlabs regionprops function, including area, perimeter, major axis length, minor axis length, solidity, eccentricity, convex area, extent, equivalent diameter, convex perimeter, fiber length (skeletonized max length), maximum inscribed radius, and maximum bleb radius (maximum secondary circle). Additional measures of form factor ($\frac{Area}{Perimeter^2}$), aspect ratio ($\frac{Majoraxislength}{Minoraxislength}$), Convexity ($\frac{Perimeter}{ConvexPerimeter}$), Curl ($\frac{Majoraxislength}{fiberlength}$), Perimeter Curl ($\frac{Perimeter}{\pi}(1-\sqrt{1-4\pi formfactor})$), Sphericity ($\frac{2*MaxInscribedRadius}{MajorAxisLength}$), Inscribed Area ($\frac{MajorAxisLength^2*\pi}{MaxInscribedRadius}$), and Bleb Ratio ($\frac{MaxBlebRadius}{MaxInscribedRadius}$) are subsequently calculated, totalling to 21 geometric measures. Collectively, this is shown schematically in figure S1.

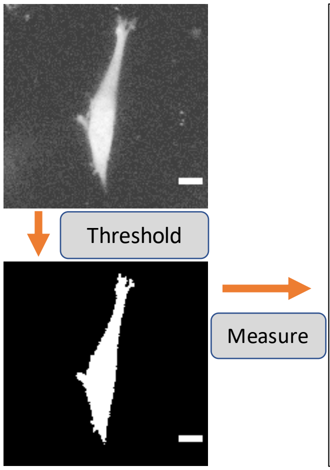

MEASURE	Quantity	
Area	490.81	μm^2
Major Axis Length	65.47	μm
Minor Axis Length	12.53	μm
Eccentricity	0.98	
Convex Area	768.30	μm^2
Equivalent Diameter	25.00	μm
Solidity	0.64	
Extent	0.31	
Perimeter	167.31	μm
Convex Perimeter	146.46	μm
Fiber Length	73.22	μm
Max Inscribed Radius	5.48	μm
Bleb Length	42.18	μm
Aspect Ratio	5.23	
Form Factor	0.02	
Convexity	1.14	
Perimeter Curl	10.51	μm
Curl	0.89	
Sphericity	0.17	
Inscribed Area	2455.99	μm
Rel. Bleb Length	7.69	

Figure S1: Schematic of image processing and measures taken from binary using custom Matlab and Python scripts (scale-bar = 10 μm). This figure is prepared with Mathlab R2020a (www.mathworks.com) and ImageJ (https://imagej.net).

Tracking cell position

In order to track the real-space center of the cell, we use maximum inscribed circle (MIC) of the cell image. Compared to imaging the nucleus directly, which causes phototoxicity and is prone to multinucleate staining, MIC does not require additional probes. To further demonstrate the accuracy of MIC, we compare short videos of dual labeled MDA-MB-231 cells where the GFP channel labels the cytoplasm and RFP channel labels the cell nucleus (SYTO-64, ThermoFisher). We find that MIC agrees very well with direct nucleus staining when determining the cell position, as shown in figure S2. For most of the frames, the deviation is less than 10% of the cell long axis. The root mean squared deviation is approximately 3 microns.

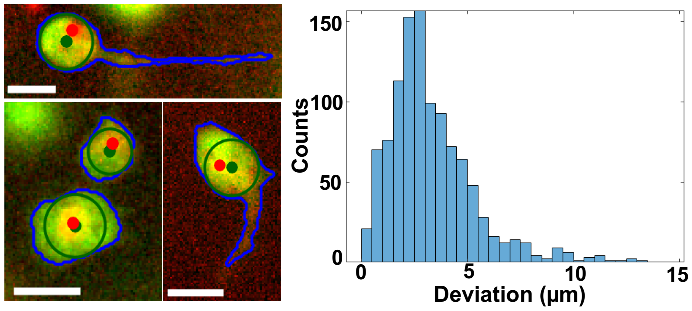

Figure S2: Positional data acquisition: Position measurements of a cell determined by the nucleus centroid stained with SYTO-64 (red) and max-inscribed circle center (green), and histogram of square deviations between the two positional measures. All scale-bars are 20 μm. This figure is prepared with Mathlab R2020a (www.mathworks.com) and ImageJ (https://imagej.net).

S3: ECM Dimension Modulates Real Space and Shape Space Dynamics

The morphodynamics of a cell display a strongly sub-diffusive characterization in geometric shape-space. Shown in Table S1, we quantify the fits of mean square displacement of measures and report the power over a ten hour lag period.

$y \sim x^n$		
Measure	n (3D)	n (2D)
Area	0.6261	0.9812
Major Axis Length	0.5761	0.8781
Minor Axis Length	0.4338	0.4702
Eccentricity	0.3638	0.3234
Convex Area	0.5738	0.9230
Equivalent Diameter	0.5999	0.9246
Solidity	0.4493	0.5794
Extent	0.4965	0.5208
Perimeter	0.5635	0.8353
Convex Perimeter	0.6043	0.9636
Fiber Length	0.5981	0.8931
Maximum-Inscribed Radius	0.5059	0.6059
Bleb Length	0.5125	0.6510
Aspect Ratio	0.4240	0.6419
Form Factor	0.4642	0.5326
Convexity	0.2645	0.4477
Perimeter Curl	0.4790	0.5681
Curl	0.2515	0.2775
Sphericity	0.5533	0.5016
Inscribed Area	0.5139	0.8617
Relative Bleb Length	0.4667	0.6052
Real-Space Migration	1.2197	1.3881

Table S1: Power (n) of anomalous diffusion quantified by fitting the mean square displacements of shape and position measures for cells embedded in a 1.5 mg/mL collagen matrix prepared at room temperature (3D) and cells plated on top of similarly prepared collagen matrices (2D).

S4: Morphological Phenotype Analysis

Details of training, validation, and test sets

Training images were processed as previously described. There were 539 raw examples in the training set made up of 120 actin-enriched leading edge, 91 filopodia, 186 bleb-based, and 141 lamellipodia examples. Importantly, we began with 101 examples of hemispherical blebbing and 85 examples of small blebbing, but the two cases were difficult to distinguish geometrically and thus combined into a single bleb-based migration state. To offset the class imbalance, a stratified training strategy was used in all models. With a shear model, 6 images were generated per raw example, with a small shear constant uniformly selected with a maximum of 0.4. Including some simulated examples of actin-enriched leading edge type, this yields 3766 examples. The training set images were acquired and classification determined by visual inspection only.

To evaluate performance, we employed the standard 10-fold cross validation scheme [2,3]. In particular, the validation set was randomly selected from available annotated training images. This means 10% of the total 3766 images, or 377 images are reserved for validation and the remaining 90%, or 3389 images were used for training per fold. In the ten-fold scheme, this process is repeated 10 times and an average of 92% accuracy is obtained. The high success rate validates the feasibility of the model. Then to obtain the final model which we will be using for experimental data analysis, all 3766 images were used for training. To examine how the trained machine learning model can be applied to real world problems, we evaluate the final performance with a dataset that is not included in the original 3766 images. This dataset is called unseen data, and the performance of the machine learning model (88% accuracy) is based on the results with this unseen dataset. This unseen test set was prepared with 50 cell images per class. The training and test sets are available on Figshare [4].

Details of machine-learning and SVM

In order to classify cells into particular migration mechanisms, we used support-vector machine (SVM) learning [5, 6]. As a maximal-margin classifier, SVM was particularly attractive as the overlap between different phenotypes was unknown. Also important is that cells can display multiple phenotypes at once and thus a soft-margin classifier was vital. Lastly, given our small dimensional space and small training-sets, SVM was an optimum choice for classification purposes.

Labeled data were first acquired as described previously. Images were binarized and then geometrical data was obtained on labeled cells for training. We performed parameter grid search for RBF, linear, and polynomial kernel models, with 10-fold cross validation to determine best performance. A grid search determined that an RBF kernel with $\gamma = 5.8^{-3}$ and $C = 4818$ yields an average training, validation, and test set accuracy of 93.1%, 86.5%, and 85.5%, respectively. However, the model generalization was improved with $\gamma = 0.01$

and $C = 1000$ (average training, validation, and test set accuracy of 92.9%, 89.8%, and 89.5%, respectively). A linear model also recorded strong performance with $C = 191.9$ with average training, validation, and test set accuracy of 90.7%, 89.4%, and 84.5%, respectively. Although easier to interpret, the linear model was found to not consistently match supervised classification. For this reason, along with the slight increase in performance, we proceeded with the RBF kernel SVM model. The final optimized SVM model used in this work performs at 92.3%, 91.8%, and 88.0% on the train, validation, and held out test sets discussed in the Random-Forest classifier.

By closely examining the misclassified held out test data sets, we notice that the most errors come from binarization process which fail to preserve the geometric features of small protrusions. Therefore the performance of the SVM classifier can be further optimized by improving imaging quality, as well as more sophisticated binarization algorithms.

Errors introduced by 2D projections of 3D cell

In order to quantify classification errors made by our SVM algorithm as a result of using geometries of 2D maximum projections of cells, we have taken high resolution confocal stacks of two sample cells, one clearly a filopodia morphology, and another clearly a blebbing cell. Using the confocal stacks, we construct deconvoluted 3D volumetric cell renderings. We numerically rotate the 3D volumetric rendered cell images at 325 uniformly spaced spherical angles (3D orientations). For each orientation, we take 2D maximum projection, and examine the classification of the projected image. For the filopodial cell, we find that the orientation introduces no more than an 8.33% classification error rate, where all the errors are made as highly elongated filopodia become oriented parallel to the viewing angle. Figure S3 shows some of the 2D projections along with visual representations of the classification error.

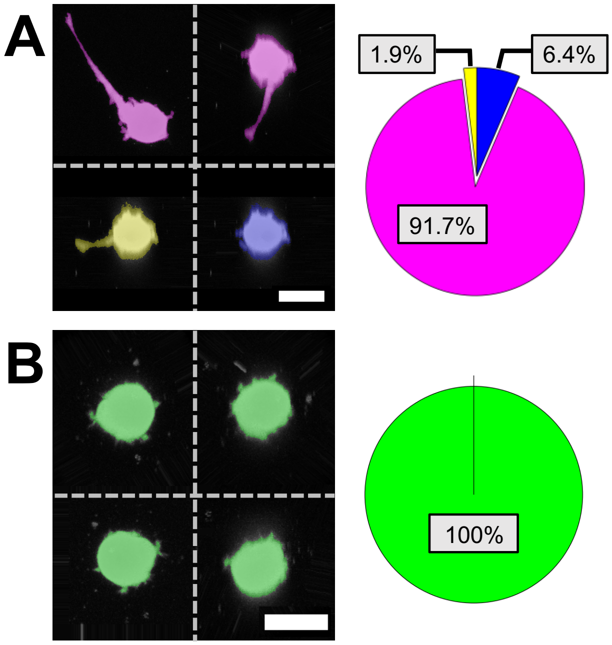

Figure S3: 2-D maximum projection images from four random orientations of cells. (A) Random views of filopodial cell and pie chart showing percentage of SVM classification over all projection angles of the same cell. (B) Random views of blebbing type cell and pie chart showing all SVM classifications of the cell were bleb-based, regardless of projection angle. Maximum projections were taken every 15 degree rotations both in θ and ϕ directions. Color represents the morphological phenotype (Actin-enriched leading edge: yellow, Filopodia: magenta, Blebbing: green, Lamellipodia: blue). Cells were imaged at 0.5 μm steps. Scale bars: 20 μm. This figure is prepared with Mathlab R2020a (www.mathworks.com) and ImageJ (https://imagej.net).

Comparisons with Random-Forest classifier

To compare performance to other multi-classification models, we have trained a Random-Forest classifier, tuning hyperparameters of depth, number of estimators, and features by grid search [7]. The optimized model uses 1200 estimators, with a maximum depth of 180 splits, and uses no feature subset selection, yielding a bagged ensemble of regression trees. We perform 10-fold cross validation on the SVM training data, utilizing class weighting to account for the class imbalance during training. We also include our 200 image held out test set for evaluation. The mean accuracy score was 83.3 7.6%, with average recall scores of 100 and 74.5% on train and held out test sets. SVM and Random-Forest classifiers generally agree very well in terms of predictions, although SVM performs notably better on the held-out test set. The Random-Forest classifier disagrees

with the SVM classifier on 294/3766 training examples (7.8%), and 34/200 test set examples (17.0%).

Feature importance

In order to interpret the features used by the SVM and random-forest classifiers in their classifications, we have examined the overall feature importance. Figure S4A shows the top two positive and negative features of each class used by the linear kernel model of SVM. Figure S4B shows the relative importance of features used by the random-forest classifier.

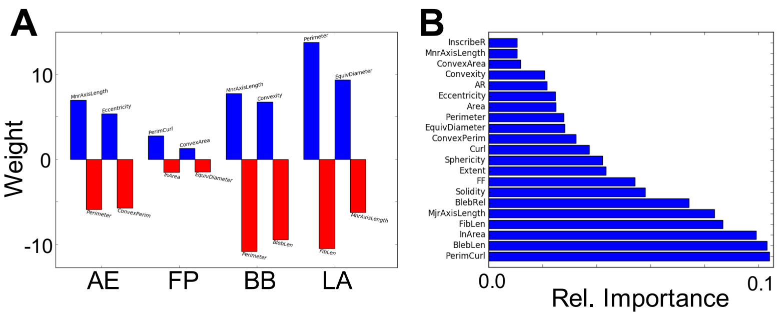

Figure S4: Top-2 linear kernel SVM class features and ordered relative importance of features in random-forest algorithm. (A) Top-2 positive and negative features used in SVM classifier from each migration phenotype (AE: Actin-Enriched Leading Edge. FP: Filopodia. BB: Blebbing-Based. LA: Lamellipodia). (B) Relative importance of features used to determine classification in random forest classifier. Feature acronyms on the y-axis are described in order top to bottom: maximum inscribed radius, minor axis length, convex area, convexity, aspect ratio, eccentricity, area, perimeter, equivalent diameter, convex perimeter, curl, sphericity, extent, form factor, solidity, relative bleb length, major axis length, fiber length, inscribed area, bleb length, and perimeter curl. This figure is prepared with Mathlab R2020a (www.mathworks.com).

Details of parametric t-SNE embedding algorithm

To visualize high-dimensional morphological trajectories in three-dimensional space, a t-Distributed Stochastic Neighbor Embedding (t-SNE) algorithm is prepared for dimensionality reduction utilizing the geometric characterization of cells without requiring labels [8]. Since the t-SNE algorithm is a non-parametric model, it utilizes all available data to separate data clusters and cannot classify new data points without retraining which often leads to different cluster shapes. We required that the non-linear algorithm could embed new data points or entire trajectories into a consistent 3D space so that different experiments could be

tracked similarly. To this end, a parametric t-SNE model is prepared which does not require re-training for new data and thus forms a consistent manifold. To make the parametric t-SNE, we train a neural network on 15,000 data points to learn a mapping by minimizing the Kullback-Leibler (KL) divergence between the Gaussian distance metric in the 21-dimensional geometric space and the Students-t distributed distance metric in the output 3-dimensional space. We use the same architecture as [9], which is a dense neural network with layers: 21 $\rightarrow$ 500 $\rightarrow$ 500 $\rightarrow$ 2000 $\rightarrow$ 3 where 21 is the input dimensionality of our geometric features and 3 is the output dimensionality representing the t-SNE embedded shape-space. In summary, the model is made up of three fully connected layers with subsequent ReLU activation after each layer, and an additional final fully connected layer calculating the three t-SNE components for each points. Functionally, the training of a non-parametric t-SNE and a parametric t-SNE is very similar. However, because the parametric model learns weights in the dense layers to perform the mapping from high to low dimensions, new input data can be transformed to the same embedded space. This model uses an Adam optimizer set to minimize the Kullback-Leibler divergence loss, and is trained with 800 iterations with a batch size of 256 examples and the tunable perplexity parameter set to 30.0. We then use the parametric t-SNE model to transform the high-dimensional trajectories of new cells into the three-dimensional space.

As seen in Fig. 2C of the manuscript, 15,000 randomly selected cell geometries were used as a training set for our parametric t-SNE model. Since the data is made up of a diverse group of cell geometries, t-SNE forms a continuous spectrum of cell shape which we call embedded "shape-space". However, once the data points are colorized using independent classification from SVM, it is obvious that the embedding is consistent with the SVM classification. Further, we use this same embedded shape-space to embed new data points and trajectories, as seen in Fig. S6.

To visualize the SVM boundaries of our training set, we also performed t-SNE on our SVM training set. Figure S5 shows cluster formation after 300 iterations with a perplexity factor of 30. Blebbing-based (green) and filopodial (magenta) morphologies show a clear separation in the t-SNE manifold both in 2-D and 3-D projections, with the space between them filled by actin-enriched leading edge (yellow) and lamellipodial migration which display some overlap but a separation in the 2-D projection.

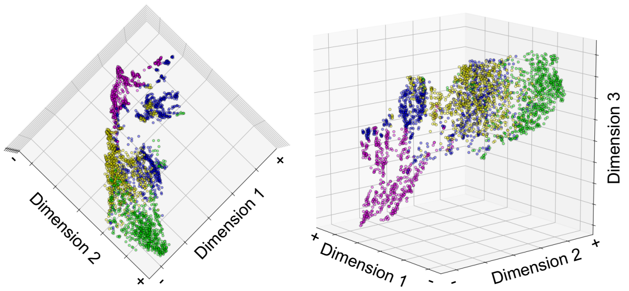

Figure S5: A 2-D projection from z-axis (left) and 3-D projection (right) of t-distributed stochastic neighborhood embedding of SVM training set. This figure is prepared with Mathlab R2020a (www.mathworks.com).

S5: Manipulating Rho/Rock Signaling by Pharmacological Treatments

t-SNE trajectories of pharmacological treatments

To demonstrate the effects of the Rho-ROCK pathway on cellular morphology, we chemically induce activation and inhibition of pathway proteins. Y27632, a specific inhibitor of Rho-associated protein kinases as well as ROCK-II activity [10–12], was purchased from Sigma-Aldrich and diluted to a working concentration at 3 μg/mL [10 μM] (0.1% v/v DMSO) in serum-free growth medium. Similarly, Rho activator II (CN03), known to robustly increase the level of GTP bound RhoA [13,14], was purchased from Cytoskeleton and diluted to a working concentration at 2 μg/mL (0.1% v/v DMSO) in serum-free growth medium.

Samples are prepared by suspending GFP-labeled MDA-MB-231 cells at low density in ice-cold neutralized collagen solution to approximately 650 cells/μL, following the same preparation and neutralization procedure described in S1. Ice-cold cell-collagen solution was then plated on a 35 mm collagen coated glass bottom dish with a 7 mm microwell diameter (MatTek, US). The microwell containing ice-cold cell-collagen solution is covered with a coverslip so that the dish may be inverted during gelation to ensure dispersion of cells in 3D. The dish is then incubated in a tissue culture incubator (37°C, 5% CO_2) for 30 minutes in order to solidify the matrix. The coverslip is removed after gelation time and the cellularized ECM is then immersed with serum-free culture medium and continuously incubated for 20 hours before imaging. Culture media is replaced with serum-free prepared growth media containing HEPES (0.1% v/v DMSO) with or without chemical dilution for experimental or control condition, respectively, and then imaged with confocal microscopy for 24 hours.

Figure S6 shows additional trajectories of cells that responded to chemical treatment. Cells treated with ROCK-inhibitor Y27632 show characteristic production of protrusions and/or sustained pre-existing protrusions. In Fig. S6A,

this is shown by most trajectories moving toward filopodial migration (magenta) or toward actin-enriched leading edge migration (yellow). Conversely, cells treated with CN03 exhibit morphologies characteristic of cell contraction. Notably, cells do not produce protrusions following treatment and typically retract protrusions post-treatment. Visually, this is seen in Fig. S6B as a notable slide toward the blebbing migration mode (green) or toward lamellipodial cell-spreading (blue). For comparison, Fig. S6C shows trajectories of cells without chemical treatment as control conditions. These cells exhibit all types of migration modes, with switching being non-uniform.

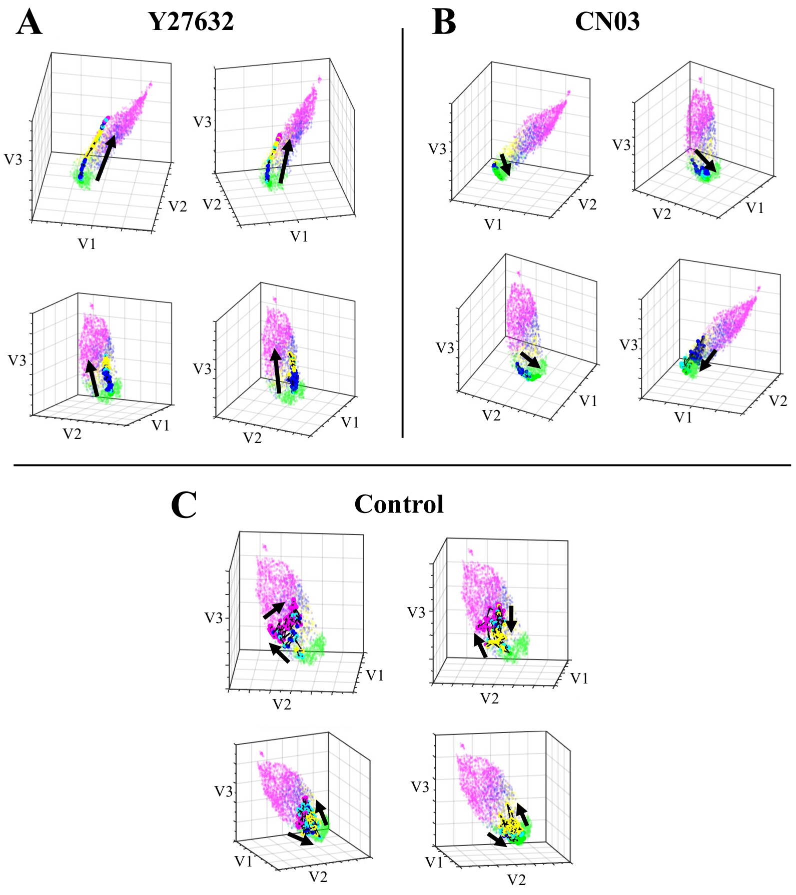

Figure S6: Additional t-SNE time projections (solid) of (A) ROCK-inhibiting Y27632, (B) Rho-activating CN03 drug treated cells, and (C) control condition non-treated cells. This figure is prepared with Mathlab R2020a (www.mathworks.com).

We have further quantified the trajectories of drug treated and control cells in the t-SNE space. In particular, we calculate the ratio between net displacement and contour length in the t-SNE space for cells with and without drug treatments. A lower value of the ratio indicates the cell makes many detours (fluctuations in direction). As shown in Fig. S7, the ratio for CN03-treated cells is about 50% lower compared with the cells treated with Y27632. This is consistent with the observation made in the main text that cells treated with CN03 manifest strongly fluctuating and diverging trajectories.

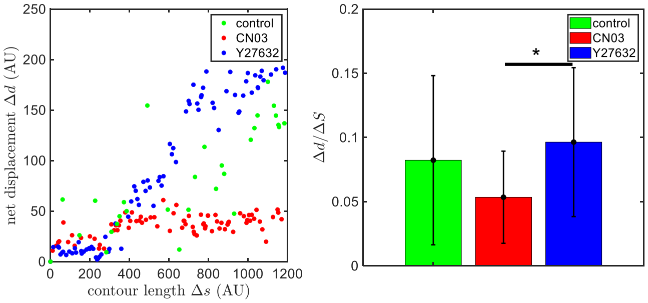

Figure S7: (A) Sample trajectories in the t-SNE space as represented as contour length versus net displacement. (B) The ratio of net displacement (Δd) and contour length (Δs) calculated when contour length first reaches 1200. The bars and error bars show means and standard deviations of around 10 sample trajectories for each condition. *: $p < 0.05$ with t-test. This figure is prepared with Mathlab R2020a (www.mathworks.com).

Cell culture and maintenance for ROCK activity and RhoA activation assays

MDA-MB-231 cells are cultured to 90% confluency (treatment and control) in 100 mm dishes for treatment. CN03-treated and control cells are first serum-starved for 24 hours, while Y27632-treated and control cells are kept in 10% FBS culture medium prior to treatment. Control condition dishes are then immersed in their respective culture medium conditions with 0.1% DMSO v/v. CN03-treated cells are immersed in serum-free culture media at 2 μg/mL CN03 with 0.1% DMSO v/v. CN03-treated and control cells are kept in a tissue culture incubator (37°C, 5% CO_2) for 4 hours. Y27632-treated cells are immersed in 10% FBS culture medium with 10 μM Y27632 and 0.1% DMSO v/v and kept in a tissue culture incubator for 12 hours. Thereafter, the media is aspirated from the dishes, and cells are washed with ice-cold PBS twice. 1 ml of cell lysis buffer with protease inhibitors and PMSF is added to the cells and the dishes are kept on ice for 20 minutes, following which the cells are transferred to a microcentrifuge tube. These cell lysates are then cleared by centrifugation at 14,000 g for 10-15 minutes at 4°C. Control and treatment lysates are then appropriately diluted to equalize protein concentration as measured by NanoDrop.

RhoA activation assay

The examination of RhoA levels is performed using a RhoA Activation Assay Kit (Cytoskeleton, Inc., Denver, CO, USA, Cat no. BK036). For RhoA pulldown, Rhotekin RBD Agarose beads are added to all the samples and incubated at 4°C for 1 hour with gentle agitation. After incubation, the beads are centrifuged, and the supernatant is removed. These beads are then washed thrice before resuspension in sample buffer for a subsequent immunoblotting procedure.

Quantitative assay for Rho kinase activity

Measurement of Rho kinase (ROCK) activity is performed using a ROCK Activity Immunoblot kit (Cell Biolabs, Inc., San Diego, CA, USA, Cat no. STA-415) according to the manufacturer's instructions. Briefly, cells resuspended in lysis buffer with protease inhibitors and PMSF are centrifuged at 14,000 g for 10 minutes at 4°C. For both, control and treatment samples, kinase reaction is initiated by the addition of a kinase/ATP/substrate solution, followed by gentle agitation at 30°C for 60 minutes. This reaction was quenched by the addition of sample buffer, after which samples are run on an SDS-PAGE gel for western blotting.

SDS-PAGE and immunoblotting

Prepared samples are separated by SDS-PAGE using a 4−20% Mini-PROTEAN TGX Precast Protein Gels (Bio-Rad Laboratories, Inc, USA) and transferred onto a Nitrocellulose membrane (Thermo Fisher Scientific, Inc., Cat no. 88018). The nitrocellulose membranes with proteins transferred over are blocked with 5% non-fat dry milk for 1 h at room temperature. The primary antibodies are then dissolved in 5% non-fat dry milk at 1:1000 concentration and used to detect the target protein in the blots. For the RhoA activation blot, the RhoA primary antibody was replaced with Anti-Mouse RhoA (LSBio, Cat. # C355184). The blots remain incubated at room temperature for 2 hours. These blots are then washed with TBST thrice for 5 minutes. Next, the blots are incubated with Goat anti-Mouse secondary antibodies at 1:2000 concentration for 1 hour at room temperature. The blots are developed using the Radiance Chemiluminescent substrate (Azure Biosystems, California, USA) and are visualized on an Azure Western blot molecular imaging system (Azure Biosystems, California, USA).

F-Actin structures respond to Rho/ROCK modulation

To visualize changes in stress fiber formation due to pharmological treatments, cells are plated on a 35 mm collagen coated glass bottom dish with a 7 mm microwell diameter (MatTek, US) at very low cell density. The dish is then incubated in a tissue culture incubator (37°C, 5% CO_2) in growth media with 10% FBS until cells reach approximately 30% confluence. Cells are then administered with pharmological treatment as follows: ROCK-Inhibitor Y27632-treated

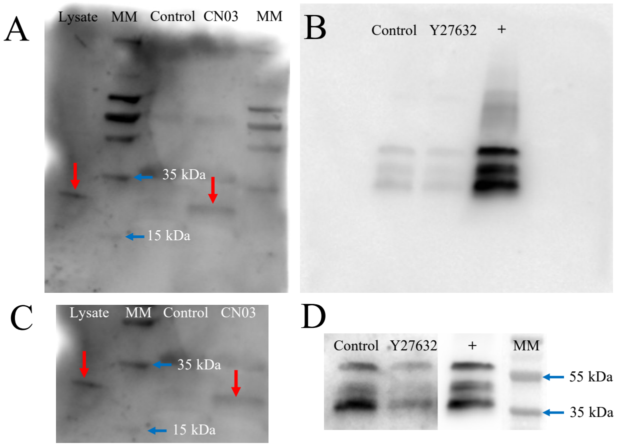

Figure S8: (A) RhoA activity immunoblot results. In lane 1 (Lysate), cellular control lysate is loaded into the gel lane representing total RhoA present. In lanes 2 and 5 (MM), a molecular marker is loaded. Lanes 3 (Control) and 4 (CN03) are loaded with non-treated and CN03-treated cell lysates following RhoA pulldown assay, respectively. Red arrows indicate RhoA band, illustrating activated RhoA from CN03-treatment in lane 4 in comparison to lane 3. (B) ROCK activity immunoblot results. Lanes 1 (Control) and 2 (Y27632) are loaded with non-treated and Y27632-treated cell lysates following kinase assay, respectively. Lane 3 (+) is loaded with purified ROCK-II used as a positive control. (C) Cropped RhoA activity immunoblot result to highlight RhoA band location. (D) Cropped ROCK activity immunoblot result. Lane 4 (MM) is loaded with a molecular marker, which is automatically overlaid by software from Azure imaging system. Less intense bands seen in lane 2 as compared to lane 1 indicate inhibition of ROCK by Y27632.

cells are immersed in growth media with 10% FBS at 10 μM Y27632 (0.1% v/v DMSO). Y27632-control comparison cells are immersed in growth media with 10% FBS (0.1% v/v DMSO). Rho-activated CN03 cells are first washed once with PBS and then serum-starved in serum-free growth media for an additional 24 hours. CN03-treated cells are then immersed in 2 μg/mL CN03 (0.1% v/v DMSO) in serum-free growth medium for 4 hours. CN03-control comparison cells are also first washed once with PBS and then serum-starved for 24 hours, before finally being immersed in new serum-free growth media (0.1% v/v DMSO) for four hours. Cells are then fixed, permeabilized, blocked, stained, and imaged following the procedure described in S1 using phalloidin (ThermoFisher).

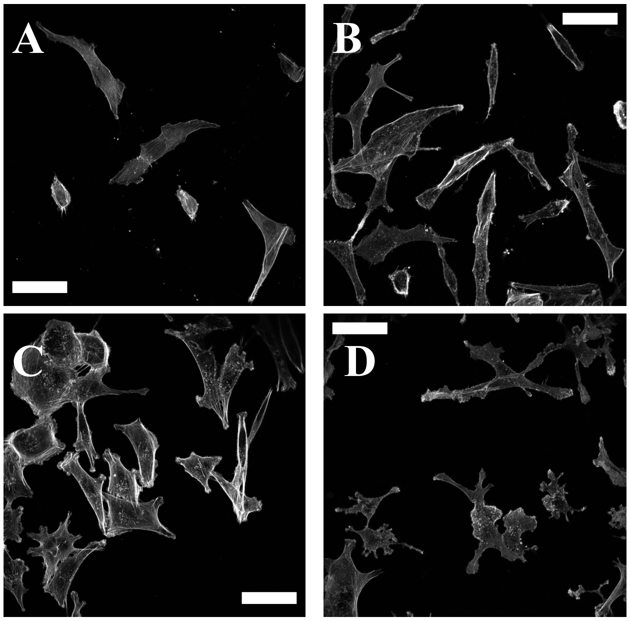

Figure S9: F-Actin staining following fixation after pharmaceutical treatment of cells for four hours. (A) CN03-control serum-starved cells (0.1% DMSO v/v) show a minimal level of stress fibers following serum starvation. (B) Upon treatment with Rho-activator CN03 (2 μg/mL, 0.1% DMSO v/v), cells present with an increase of active stress fibers. (C) Y27632-control cells cultured in 10% FBS (0.1% DMSO v/v) display a high degree of stress fibers which are rapidly eliminated following (D) treatment with ROCK-inhibitor Y27632 (10 μM, 0.1% DMSO v/v). Scale-bars = 50 μm.

S6: ECM Characterization

Confocal reflection intensity autocorrelation function

In order to quantify the density fluctuations of collagen ECM, we compute the autocorrelation functions of confocal reflection images of collagen gels. Reflection images are first background subtracted using a rolling ball with radius of 50 pixels (26.88 μm). Images are then log-transformed to make fibers highly quantifiable. Images are then mean subtracted, and the autocorrelation is calculated. Following, the autocorrelation is normalized and then smoothed using interpolation. The results are shown in figure S10. The spatial uniformity indicates an appropriate distribution of randomly oriented gel fibers. Additionally, these show that the decay in the autocorrelation is slower for higher density gels, an indication of the fiber quantity, and much faster for higher temperature gel, caused by the shorter fiber lengths and smaller pores [15]. The anisotropy of decay in the final graph indicates the direction of alignment.

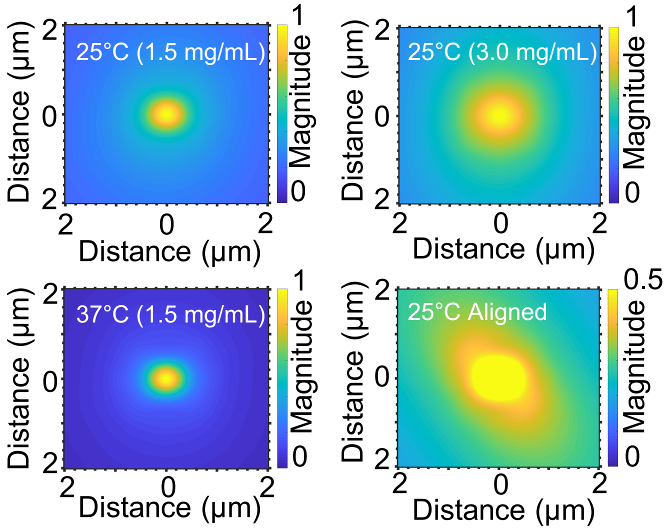

Figure S10: Autocorrelations shown for 25°C 1.5 mg/mL Randomly oriented gel (upper left), 25°C 3.0 mg/mL Randomly oriented gel (upper right), 37°C 1.5 mg/mL Randomly oriented gel (lower left), and 25°C 1.5 mg/mL Aligned oriented gel (lower right). The magnitude of the aligned gel differs so that the anisotropy can be clearly observed in comparison to the room temperature gel. This figure is prepared with Mathlab R2020a (www.mathworks.com).

ECM fiber network coherency

In order to determine the degree of local and global alignment of collagen fibers, we take the pre-processed confocal reflection images and use OrientationJ with 9-14 circular ROIs per image packed without overlap, with ROI sizes ranging from 145 to 450 pixel diameter. At least 3 images (one per experiment) were used to quantify coherency. Empirically, larger ROIs are reliable to quantify global alignment, while smaller ROIs were better used for local alignment measures. ROIs smaller than 145 pixels are not used, as it was noticed the coherency measured by OrientationJ can be highly biased for large fibers such as in 25°C gels (145 pixels [78 μm] is approximately twice the average fiber length [$\approx$ 41 μm] in a 25°C gel). The results are shown in figure S11 and follows previous literature [16].

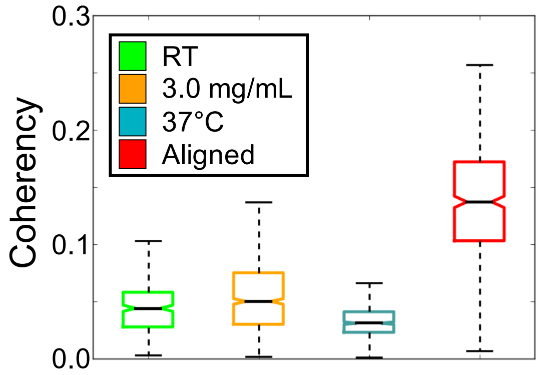

Figure S11: Bar and whisker plot of coherency measurements taken using custom Matlab scripts utilizing OrientationJ plug-in for ImageJ (NIH). Measurements of back-reflection collagen images using circular ROIs ranging from 145 to 450 pixel (80 - 240 μm) diameter for differing ECM architectures. RT: 1.5 mg/mL 25°C gel, 3.0 mg/mL: 3.0 mg/mL 25°C gel, 37°C: 1.5 mg/mL 37°C gel, Aligned: 1.5 mg/mL 25°C gel prepared using the alignment protocol. Whiskers extend to 1.5 times the inter-quartile range. Utilizing a multiple comparison test with the Bonferroni method, all groups are significantly different from each other (RT vs. 3.0 mg/mL: $p < 0.05$. All other comparisons: $p < 0.01$.) This figure is prepared with Mathlab R2020a (www.mathworks.com).

Rheology

Strain sweep rheology measurements are performed on varying density gels and varying gelation temperature with a AR-G2 rheometer (TA instruments) at a 1 Hz frequency in a parallel plate geometry. A standard peltier plate (TA instruments) allows gels to be formed at 25°C and 37°C. Young's modulus is shown in table S2. As shown, storage moduli in the linear regime for gels with 1.5, and 3.0 mg/mL collagen prepared at 25°C are about 325 and 830 Pa, respectively. Similarly, for gels with 1.5, and 3.0 mg/mL collagen prepared at 37°C are about 170 and 425 Pa, respectively.

**Linear Regime G'**		
Collagen Density	Temperature	G'
1.5 mg/mL	25°C	325 Pa
1.5 mg/mL	37°C	171 Pa
3.0 mg/mL	25°C	832 Pa
3.0 mg/mL	37°C	427 Pa

Table S2: The linear storage modulus of collagen fiber networks using AR-G2 Rheometer (TA Instruments) in a parallel plate geometry at a 1 Hz frequency.

S7: Additional Details of Morphological Phenotype Dynamics

Dwell time definition

The dwell time of state i is determined by using

$$D_{i\to i} = \frac{1}{1 - r_{i\to i}}.$$

where $r_{i\to i}$ is the transition rate from state i back to itself, and $D_{i\to i}$ is the corresponding dwell time. We find that this definition of dwell time maximizes the usefulness of data and is more robust to experimental shortfalls that affect the naive method of simply counting the number of frames a cell remains in the same phenotype for.

Details of transition rate calculations

Assuming the probability distribution of cell morphological phenotypes follow a Boltzmann distribution, then the probability P_i is given by

$$P_i = \frac{1}{1 + \sum_{j\neq i}^{N} \frac{r_{i\to j}}{r_{j\to i}}}$$

where N is the number of states, $r_{i\to j}$ is the transition rate from state i to state j, and conversely $r_{j\to i}$ is the transition rate from state j to state i. The transition matrix is thus calculated as probability per unit time, and hence each row in the transition matrix will sum to 1

$$\sum_{j}^{N} r_{i\to j} = 1.$$

The transition rate $r_{i\to j}$ is defined as the probability of transition from state i to state j per unit time, given by

$$r_{i\to j} = \frac{\sum_{m}^{N_{i\to j}} \frac{1}{dt_{i\to j}^{m}}}{\sum_{k}^{C} \sum_{m}^{N_{i\to k}} \frac{1}{dt_{i\to k}^{m}}}$$

where $dt_{i\to j}^{m}$ is the transition time of the mth observation from class i to class j, C is the number of classes, and $N_{i\to j}$ is the number of observed transitions from class i to class j. The transition time $dt_{i\to j}^{m}$ is the number of frames until a cell goes from a state with classification probability above threshold (>60%) to another state that exceeds the threshold, and therefore has a lower bound of 1 if the transition is immediate and increases as the cell passes through intermediate states. Using this method, we find that the transition rates and dwell times are stable, regardless of the length of trajectories in computational experiments.

Following SVM classification, phenotype dynamics can be properly drawn from data. Importantly, where maximum decisions by the SVM classifier do not exceed 60%, the classification is thus determined to an intermediate between two states. The intermediate state can be a chimera of two states, with most occurrences being as a cell transitions between morphological phenotypes. Because this state is not considered to be unique, we calculate the transition rate from state i that passes through N intermediate states prior to state j as $1/N_{frames}$. We find by simulation that this method can most-accurately recover transition rates in comparison to methods using soft-max or ignoring intermediate states.

Probability flux

To investigate broken detailed balance in morphological phenotype space, we report probability flux calculations shown in figure S12. The probability flux from state i to state j is given by

$$\Delta\phi_{i\to j} = \frac{N_{i\to j}}{\sum_k \sum_l N_{k\to l}}$$

where $N_{i\to j}$ is the number of transitions from state i to state j, and both summations in the denominator are over all available states. The probability flux $\phi_{i\to j}$ and the transition rate $r_{i\to j}$ are related by $\phi_{i\to j} = P_i r_{i\to j}$, where P_i is the probability of a cell being at state i. The net probability flux between states i and j is given by $\Delta\phi_{i\to j} - \Delta\phi_{j\to i}$. We find that the net flux between states are all less than 0.003 probability difference per hour for cells in any ECM condition we tested. To reveal if any small difference in probability flux may be significant, we also report the probability flux percent difference between states i and j is given by

$$\frac{\Delta\phi_{i\to j} - \Delta\phi_{j\to i}}{\Delta\phi_{i\to j} + \Delta\phi_{j\to i}}$$

We find that the maximum net probability flux percent difference for cells in any ECM condition evaluated is less than 9%.

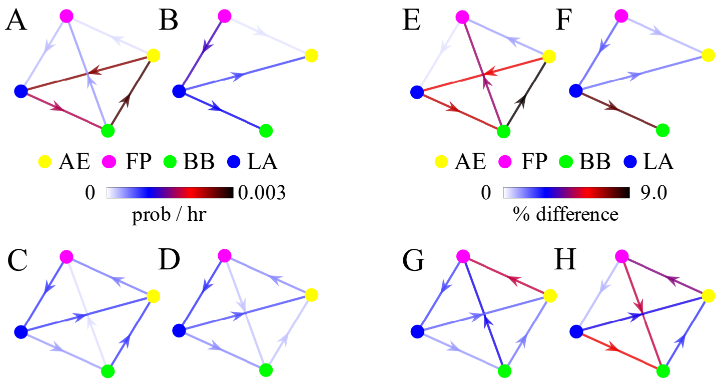

Figure S12: (A-D) Net probability flux calculations under varying ECM conditions. (E-H) Percent difference of net probability flux under varying ECM conditions. (A, E) collagen ECM prepared at room temperature (RT) and [*col*] = 1.5 mg/mL. (B, F) collagen ECM prepared at RT and [*col*] = 3.0 mg/mL. (C, G) collagen ECM prepared at 37 °C and [*col*] = 1.5 mg/mL. (D, H) collagen ECM prepared at RT and [*col*] = 1.5 mg/mL with flow-aligned collagen fibers. This figure is prepared with Mathlab R2020a (www.mathworks.com).

S8: Motility Analysis of Morphological Phenotypes

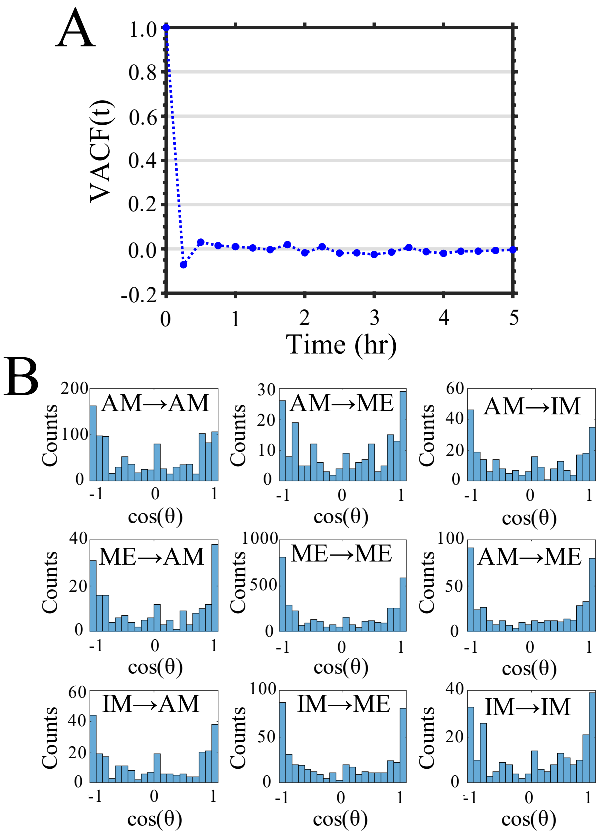

Figure S13: (A) Velocity autocorrelation and (B) Direction change given by $cos(\theta)$ between consecutive velocity vectors for cell migration as cells switch phenotypes. AM: Amoeboid, ME: Mesenchymal, IM: Intermediate. This figure is prepared with Mathlab R2020a (www.mathworks.com).

In order to determine the migrational persistence of migration modes, we report first the velocity autocorrelation observed for cells in collagen ECM prepared at room temperature and $[col] = 1.5$ mg/mL. We find that the autocorrelation quickly decays to zero in a single time step (15 minutes) shown in figure S13(A). We additionally checked the $cos(\theta)$ distribution between consecutive steps and find that the concurrent steps seem to be taken randomly in direction, as given by the U-shaped distribution of figure S13(B).

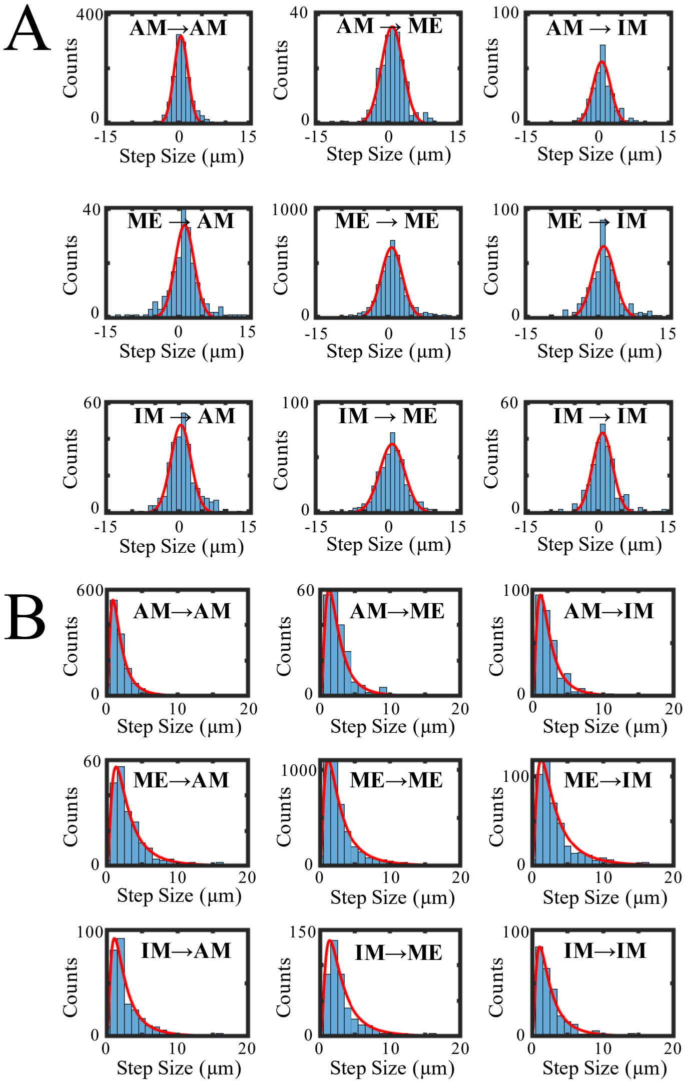

Figure S14: (A) Gaussian fitting (red) of experimentally measured (blue) step size (displacement in one frame, or 30 minutes) distributions in the persistent direction (bin width = 1 μm) for various migration mode transitions. (B) Fit (red) of experimentally measured binned (blue) step size magnitude distributions using log-normal distribution. This figure is prepared with Mathlab R2020a (www.mathworks.com).

To evaluate the motility of various migration mode transitions, steps sizes (frame-to-frame displacements) are separated into components parallel and per-

pendicular to the direction of the previous step. Figure S14A shows the binned step size distribution in the persistent direction fit with a Gaussian distribution. It can be seen that these fits miss a significant portion of large steps. Rather, we find more suitable fits are obtained by fitting the magnitude of the step sizes, which follow a log-normal distribution as shown in figure S14B. The parameters from fits shown in figure S14B are subject to bin-size, and therefore we instead report parameters obtained by fitting the empirical cumulative distribution (CDF) of step size magnitude with a log-normal CDF, shown in figure S15.

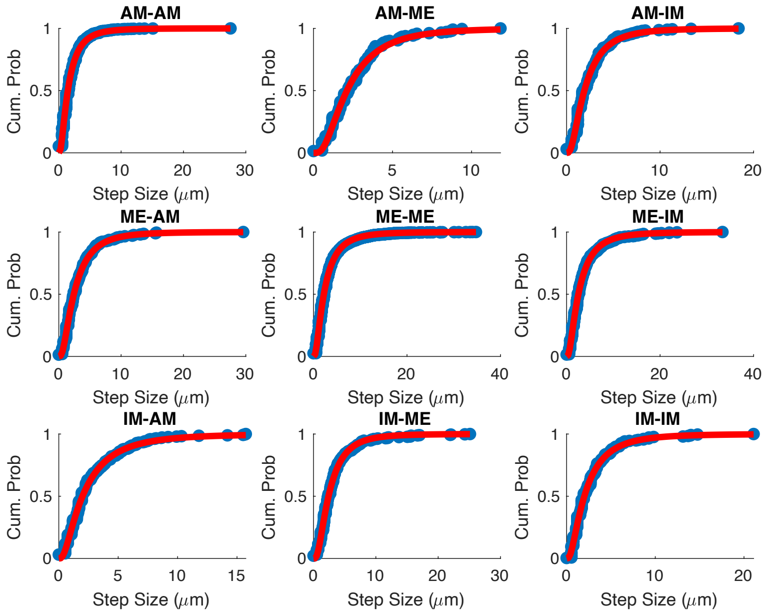

Figure S15: Empirical cumulative distribution of experimentally measured step size magnitudes (blue) and log-normal function fits (red) for dwell and transitions between amoeboid (AM), mesenchymal (ME), and intermediate (IM) modes. This figure is prepared with Mathlab R2020a (www.mathworks.com).

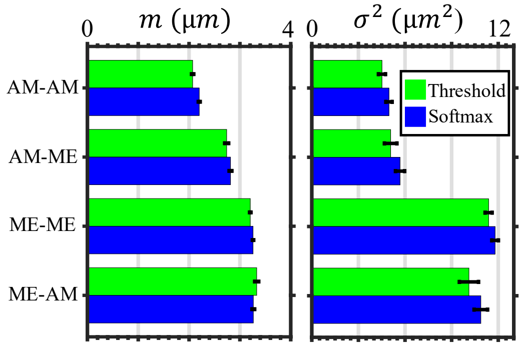

Figure S16: Motility characteristics depend on the morphological phenotype transitions. The bar plots show the means and variances by fitting cell step magnitudes with a log-normal distribution. Threshold: the morphological phenotypes are determined as in the main text. Cells with classification scores less than 60% are considered in the intermediate state. Softmax: the intermediate state is reclassified as AM or ME state based on the most likely phenotype. Error bars show the 95% confidence interval of fitted parameters. This figure is prepared with Mathlab R2020a (www.mathworks.com).

Because intermediate states account for a sizable portion of the data (approximately 10%), we seek to further evaluate its impacts in the motility characteristics. As an alternative approach, we eliminate the intermediate state by classifying cells according to their highest probability scores. This reduces the number of coarse-grained phenotypes from three (AM, ME and intermediate) to two (AM and ME). Using this approach, we reanalyze the motility characteristics and find there are little changes in the fitted means and variances of the steps. Therefore neither the intermediate states, nor the precise value of classification threshold (currently 60%) contribute significantly to the results presented in Fig. 5.

In our study, to reduce the imaging phototoxicity, and to account for the high resolution single cell images that vary their shapes, we uniformly apply the same MIC calculation to all cells, regardless of the cell shape. We also conduct additional analysis to examine the relation between MIC cell center and cell shape. For instance, we find the step size normalized by cell aspect ratio have similar distributions when tracked via MIC or cell nucleus (NUC for short) (figure S17A). Since aspect ratio is one of the most significant features that distinguish AM and ME cells, we conclude that MIC and NUC tracking yield consistent relations between cell motility and cell geometry.

We also confirm the conclusion that ME cells are more motile than AM cells when tracking cell nucleus or by tracking center of mass (CENT for short) (figure S17B-D). These results are consistent with our findings in the main text (Fig. 5).

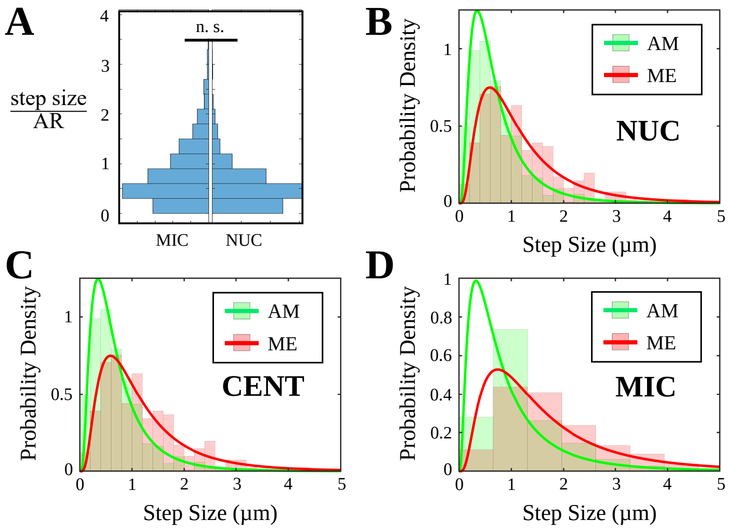

Figure S17: (A) The histogram of step sizes normalized by cell aspect ratio. MIC: tracking cells using maximum included circle, NUC: tracking using center of cell nuclues. Kolmogorov-Smirnov test shows these two distributions are statistically the same. (B,C,D) Step sizes of MDA-MB-231 cells tracked using (B) center of mass of cell nuclei stained with SYTO-64, (C) center of mass of cell body, and (D) center of maximum inscribed circle. Step sizes are measured between concurrent frames (15 minutes) during live-tracking of individual cells during 3D migration in [col] = 1.5 mg/mL collagen I matrix. Shown are step sizes between dwelling events of amoeboid (AM) and mesenchymal (ME) classified cells. Transparent bars are histograms of step sizes (bin-width is 0.2 μm (B-C), and one pixel or 0.656 μm for C). Solid lines are the probability density fits of the log normal distribution with parameters determined by fitting the log normal cumulative distribution function. The Johnson Transform of the log normal mean and standard deviation parameters returns the mean step size of AM and ME as determined by tracking (B) nucleus center of mass as 0.75 μm and 1.24 μm, respectively, and variances of 0.37 μm^2 and 0.99 μm^2, respectively; by tracking (C) cell center of mass as 0.70 μm and 1.20 μm, respectively, and variances of 0.33 μm^2 and 0.10 μm^2, respectively; and by tracking (D) center of maximum inscribed circle as 0.98 μm and 1.77 μm, respectively, and variances of 1.05 μm^2 and 2.48 μm^2, respectively. Note that the MIC tracking is limited to pixel resolution, which contributes to the relatively larger uncertainties in fitting a small data set. Step sizes are determined over N_{AM} = 587 frames and N_{ME} = 205 frames while tracking 26 cells for an average trajectory length of 10.4 hours.

S9: Interface Calculations

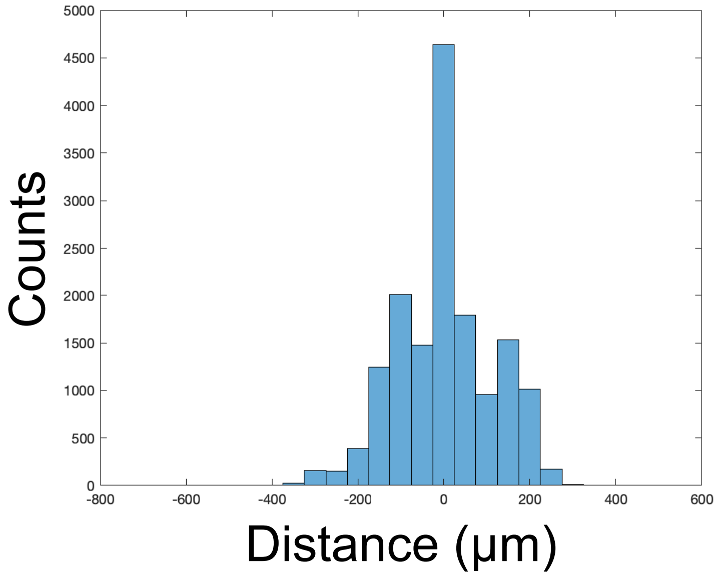

Figure S18: Frequency of cell locations away from interface (at 0). Negative is in 37°C gel, Positive indicates 25°C gel). This figure is prepared with Mathlab R2020a (www.mathworks.com).

Experiment details

Interface experiment was done in triplet. Briefly, the outer gel was first made by gelling cells in collagen solution (1.5 mg/mL neutralized) at 25°C for 20 minutes on the DIGME stage [17]. After, the needle was gently removed and a new ice-cold collagen solution (1.5 mg/mL neutralized) containing cells was then dripped into the hole, gently swirled, and then placed into the incubator at 37°C for 15 minutes. After, collagen gels were immersed in 3 mL of growth medium and continuously incubated for 24 hours before imaging. Imaging was taken near the interface, imaging bright-field, fluorescence (green), and back-reflection confocal images. Using the back-reflection images, the interface was manually traced out through the z-stack. The distance to the interface from cell centroids in corresponding z-stacks were then measured away from the closest marked interface point using Matlab bwdist. The frequency vs distance is shown in figure S18, indicating a large number of cells were images close to the interface.

Details of interface analysis

Migration mode transitions were first determined by using continuous trajectories accounting properly for intermediate state classification, and then distances away from the interface were determined by the initial state location. A 1-D Gaussian kernel was used to acquire continuous local spatial probability densities of transitions (per hour), centered every 5.376 μm (10x the distance-to-pixel ratio) with a standard deviation of 26.88 μm. This yields the probability density (per hour) of observing a transition $P(i \rightarrow j, x)$ at a location x, as used in the main text. To mitigate the bias from non-uniform cell density, we then divide the prior probability by the spatial probability of observing a cell within the spatial window centered at x, $M(x)$. $M(x)$ is calculated using the same Gaussian kernel.

S10: Actin Cytoskeleton Structures of Different Morphological Phenotypes

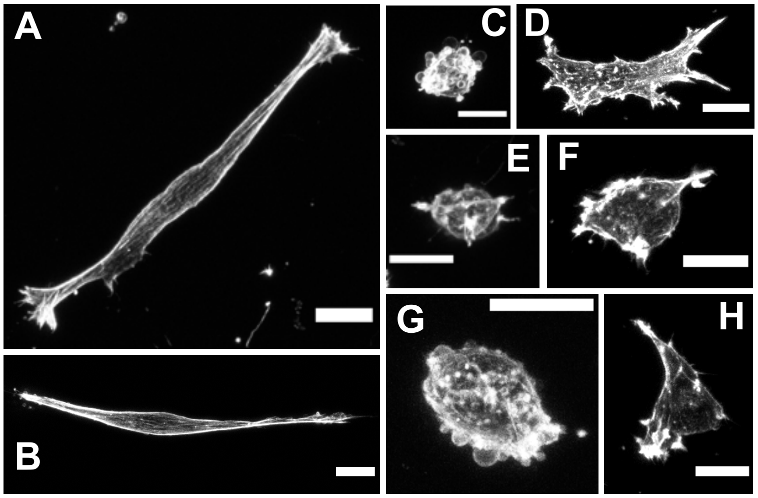

Figure S19: Actin immunostaining of MDA-MB-231 cells embedded in 3D collagen type I gel. Different morphological phenotypes exhibit structural features consistent with the corresponding migration modes. A,B: Filopodial cells. C, G: Blebbing cells. D, H: Lamellipodial cells. E, F: Actin-enriched leading edge cells.

To further demonstrate the biological significance of the machine-classified morphological phenotypes, we have examined the characteristic structures of actin cytoskeletons of different cell types. Figure S19 shows the immunofluorescent images of two typical cells per each morphological phenotype. It can be seen that filopodial cells (FP type, figure S19 A-B) have highly polarized F-actin bundles extending the long axis of the cell body. Blebbing cells (BB type, figure S19 C and G) show spherical blebs of various curvatures at cell membrane without actin polarization. Lamellipodial cells (LP type, figure S19 D and H) exhibit smooth actin-rich arcing at leading edges of overall elongated cell bodies. Finally, actin-enriched leading edge cells (AE type, figure S19 E and F) display sharp, actin-rich protrusions from the cell surface without actin polarization in the cell body. These features are consistent with previously reported actin cytoskeleton structures of different migration modes [18–21].

References

[1] Jihan Kim, Yu Zheng, Amani A. Alobaidi, Hanqing Nan, Jianxiang Tian, Yang Jiao, and Bo Sun. Geometric dependence of 3d collective cancer invasion. *Biophysical Journal*, 118(5):1177 – 1182, 2020.

[2] Sanjay Yadav and Sanyam Shukla. Analysis of k-fold cross-validation over hold-out validation on colossal datasets for quality classification. In *2016 IEEE 6th International conference on advanced computing (IACC)*, pages 78–83. IEEE, 2016.

[3] Payam Refaeilzadeh, Lei Tang, and Huan Liu. Cross-validation. *Encyclopedia of database systems*, 5:532–538, 2009.

[4] Christopher Eddy. MDA-MB-231 Morphology Training and Test Sets. *FigShare*, 10 2020. doi: 10.6084/10.6084/m9.figshare.13125086.

[5] Corinna Cortes and Vladimir N. Vapnik. Support-vector networks. *Machine Learning*, 20(3), 1995. doi: 10.1007/BF00994018.

[6] Asa Ben-Hur, David Horn, Hava T. Siegelmann, and Vladimir Vapnik. Support vector clustering. *J. Mach. Learn. Res.*, 2:125137, March 2002.

[7] Leo Breiman. Random forests. *Machine learning*, 45(1):5–32, 2001.

[8] Gordon J. Berman, Daniel M. Choi, William Bialek, and Joshua W. Shaevitz. Mapping the stereotyped behaviour of freely moving fruit flies. *Journal of The Royal Society Interface*, 11(99):20140672, 2014.

[9] Laurens Van Der Maaten. Learning a parametric embedding by preserving local structure. In *Artificial Intelligence and Statistics*, pages 384–391. PMLR, 2009.

[10] Renaud Poincloux, Olivier Collin, Floria Lizárraga, Maryse Romao, Marcel Debray, Matthieu Piel, and Philippe Chavrier. Contractility of the cell rear drives invasion of breast tumor cells in 3d matrigel. *Proceedings of the National Academy of Sciences*, 108(5), 2011. doi: 10.1073/pnas.1010396108.

[11] Simon Gordonov, Mun Kyung Hwang, Alan Wells, Frank B. Gertler, Douglas A. Lauffenburger, and Mark Bathe. Time series modeling of live-cell shape dynamics for image-based phenotypic profiling. *Integrative Biology*, 8(1), 2015. doi: 10.1039/c5ib00283d.

[12] Masahiro Matsubara and Mina J Bissell. Inhibitors of rho kinase (rock) signaling revert the malignant phenotype of breast cancer cells in 3d context. *Oncotarget*, 7(22), 2016. doi: 10.18632/oncotarget.9395.

[13] Meng-Horng Lee, Pei-Hsun Wu, Daniele Gilkes, Ivie Aifuwa, and Denis Wirtz. Normal mammary epithelial cells promote carcinoma basement membrane invasion by inducing microtubule-rich protrusions. *Oncotarget*, 6(32):32634–32645, 2015. doi: 10.18632/oncotarget.4728.

[14] Abdelkader Daoud, Udhayakumar Gopal, Jasmine Kaur, and Jennifer S Isaacs. Molecular and functional crosstalk between extracellular hsp90 and ephrin a1 signaling. *Oncotarget*, 8(63), 2017. doi: 10.18632/oncotarget.22370.

[15] Christopher Allen Rucksack Jones, Long Liang, Daniel Lin, Yang Jiao, and Bo Sun. The spatial-temporal characteristics of type i collagen-based extracellular matrix. *Soft Matter*, 10(44):8855–8863, November 2014.

[16] T. D. Clemons, M. Bradshaw, P. Toshniwal, N. Chaudhari, A. W. Stevenson, J. Lynch, M.W. Fear, F. M. Wood, and K. Swaminathan Iyer. Coherency image analysis to quantify collagen architecture: implications in scar assessment. *RSC Adv.*, 8:9661–9669, 2018.

[17] Amani A. Alobaidi and Bo Sun. Probing three-dimensional collective cancer invasion with digme. *Cancer Convergence*, 1(1), 2017. doi: 10.1186/s41236-017-0004-9.

[18] Florian Geiger, Daniel Rdiger, Stefan Zahler, and Hanna Engelke. Fiber stiffness, pore size and adhesion control migratory phenotype of mda-mb-231 cells in collagen gels. *PLOS ONE*, 14(11):1–13, 11 2019.

[19] Andrew D Doyle, Nicole Carvajal, Albert Jin, Kazue Matsumoto, and Kenneth M Yamada. Local 3d matrix microenvironment regulates cell migration through spatiotemporal dynamics of contractility-dependent adhesions. *Nature communications*, 6(1):1–15, 2015.

[20] Congying Wu, Sreeja B Asokan, Matthew E Berginski, Elizabeth M Haynes, Norman E Sharpless, Jack D Griffith, Shawn M Gomez, and James E Bear. Arp2/3 is critical for lamellipodia and response to extracellular matrix cues but is dispensable for chemotaxis. *Cell*, 148(5):973–987, 2012.

[21] Ryan J Petrie and Kenneth M Yamada. At the leading edge of three-dimensional cell migration. *Journal of cell science*, 125(24):5917–5926, 2012.
